# Automated gross tumor volume contour generation for large‐scale analysis of early‐stage lung cancer patients planned with 4D‐CT

**DOI:** 10.1002/mp.14644

**Published:** 2020-12-30

**Authors:** Angela Davey, Marcel van Herk, Corinne Faivre‐Finn, Sean Brown, Alan McWilliam

**Affiliations:** ^1^ Division of Cancer Sciences School of Medical Sciences Faculty of Biology, Medicine and Health The University of Manchester Manchester UK; ^2^ Department of Radiotherapy Related Research The Christie NHS Foundation Trust Manchester UK; ^3^ Department of Clinical Oncology The Christie NHS Foundation Trust Manchester UK

**Keywords:** 4D‐CT, GTV, iGTV, lung cancer, SABR, swept volume, tumor motion

## Abstract

**Purpose:**

Patients with early‐stage lung cancer undergoing stereotactic ablative radiotherapy receive four‐dimensional computed tomography (4D‐CT) for treatment planning. Often, an internal gross target volume (iGTV), which approximates the motion envelope of a tumor over the breathing cycle, is delineated without defining a gross tumor volume (GTV). However, the GTV volume and shape are important parameters for prognostic and dose modelling, and there is interest in radiomic features extracted from the GTV and surrounding tissue. We demonstrate and validate a method to generate the GTV from an iGTV contour to aid retrospective analysis on routine data.

**Method:**

It is possible to reconstruct the geometry of a tumor with knowledge of tumor motion and the motion envelope formed during respiration. To demonstrate this, the tumor motion path was estimated with local rigid registration, and the iGTV positioned incrementally at stations along the reverse path. It is shown that the tumor volume is the largest set common to the intersection of the iGTV at these positions, hence can be derived. This was implemented for 521 lung lesions on 4D‐CT. Eleven patients with a GTV delineation performed by a radiation oncologist on a reference phase (50%) were used for validation. The generated GTV was compared to that delineated by the expert using distance‐to‐agreement (DTA), volume, and distance between centres of mass. An overall success rate was determined by detecting registration inaccuracy and performing a quality check on the routine iGTV. For successfully generated contours, GTV volume was compared to iGTV volume in a prognostic model for overall survival.

**Results:**

For the validation dataset, DTA mean (0.79 – 1.55 mm) and standard deviation (0.68 – 1.51 mm) were comparable to expected observer variation. Difference in volume was < 5 cm^3^, and average difference in position was 1.21 mm. Deviations in shape and position were mainly caused by observer differences in iGTV and GTV interpretation as opposed to algorithm performance. For the complete dataset, an acceptable contour was generated for 94% of patients using statistical and visual assessment to detect failures. Generated GTV volumes improved prognostic model performance over iGTV volumes.

**Conclusion:**

A method to generate a GTV from an iGTV and 4D‐CT dataset was developed. This method facilitates data analysis of patients with early‐stage lung cancer treated in the routine setting, that is, data mining, prognostic modeling, and radiomics. Generation failure detection removes the need for visual assessment of all contours, reducing a time‐consuming aspect of big‐data analysis. Favorable prognostic performance of generated GTV volumes over iGTV ones demonstrates opportunities to use this methodology for future study.

## 1 INTRODUCTION

Patients with early‐stage lung cancer undergoing stereotactic ablative radiotherapy (SABR) receive four‐dimensional computed tomography (4D‐CT) for treatment planning, as advised in UK and international guidelines.^1^, ^2^ A 4D‐CT dataset typically contains ten three‐dimensional CT (3D‐CT) volumes capturing the tumor at different phases of respiration. In 4D treatment planning, the gross tumor volume (GTV) is generally not delineated, depending on the method used for dealing with patient‐specific motion.^3^ The ICRU defined internal target volume (ITV) encompasses motion in the union of clinical target volumes (CTVs) from all phases.^4^ For SABR, a CTV expansion is not applied because of high incidental dose to surrounding tissue.^5^, ^6^, ^7^ In the case of no CTV, the method to account for motion is the union of all phase GTVs, coined the internal gross target volume (iGTV). Often, to avoid contouring phase GTVs, the iGTV (or “motion‐adapted GTV”) is delineated on the maximum intensity projection (MIP) scan. This represents the tumor volume union for solid tumors surrounded by low‐density tissue.^8^, ^9^ The MIP approach incorporates both tumor volume and motion into a single delineation. Hence, for the 55% of UK SABR centres that adopt this approach, the GTV is never defined and not available for analysis of routine data.^10^

In retrospective analysis, the GTV volume and shape are important parameters for prognostic and dose modeling.^11^, ^12^ Also, radiomic features describing the GTV and surrounding tissue have been linked to local control, metastasis, and overall survival.^13^, ^14^, ^15^ So far, there are few radiomic studies using 4D‐CT data unless a GTV has been delineated within the clinical protocol,^11^, ^14^ or retrospective contouring is performed.^15^ For large datasets, manual contouring is time‐consuming, therefore a fully automated method to acquire the GTV contour is necessary. This can be obtained from the iGTV contour available in clinical datasets.

So far, Johnson et al proposed the only method to generate a GTV from an iGTV.^16^ Erosion kernels were applied independently to upper and lower lobe lung tumors, derived from average difference between iGTV and GTV for 25 tumors of varied size and location. Importantly, the training cohort had a larger average tumor size than tumors typically treated with SABR. Furthermore, early‐stage tumors display a wider range of motion variability, even when grouped by location.^17^ This motion is not directly related to volume and the exact trajectory is unique,^18^ so a personalised erosion method is required to retrospectively obtain the GTV.

For a personalised method, we show that with the motion path and total motion envelope over the respiratory cycle (iGTV) it is possible to reconstruct precise geometry of the tumor. We implemented this by positioning the iGTV at stations along the reverse motion path and formed intersections with the stationary iGTV at these locations. Since the tumor forms the iGTV over the forward trajectory it must invariably be the largest set common to these intersections with itself. This is the first time the approach has been both described and implemented for this purpose. We validated results on expert contours and demonstrated applicability to a large clinical dataset. This method will facilitate future retrospective studies in patients with early‐stage lung cancer. The intended purpose is for this contour to be used in data analysis, such as data mining and radiomics, and not in clinical practice. The use of an observer iGTV to generate the GTV is a useful tool alone or alongside alternative auto‐segmentation options as it incorporates decisions used for treatment into the segmentation process, which is important for modelling patient outcome.

## 2 MATERIALS AND METHODS

### 2.A GTV generation theory

#### 2.A.1 Kinematics of tumor motion

Before detailing the implementation of the method, we describe how tumor geometry can be obtained from the iGTV and motion path of the tumor. Both the tumor and motion envelope (iGTV) can be described as a *control volume*, that is, a volume in space that in the most general sense can deform, move, and rotate. Selecting a reference position, the tumor is denoted $V_{\mathrm{ref}}^{*}$, and all coordinates in the volume denoted, $\chi \in V_{\mathrm{ref}}^{*}$.

The tumor can be considered a rigid body, as rotation and deformation are small compared to translational motion.^19^ Therefore, the tumor at each phase of respiration, is $V_{\mathrm{ref}}^{*}$ at a position, described by k=0%,10%,…,90%. The motion path, $\gamma_{\chi }\left(t\right)$, is then only dependent on the reference coordinates and time. Assuming the tumor motion consists of piecewise linear trajectories to each phase position, the motion envelope is defined as the set $V_{\mathrm{env}}=\left\{\gamma_{\chi }\left(t\right):\forall \chi \in V_{\mathrm{ref}}^{*},\forall t\in \left[t_{0\%},t_{90\%}\right]\right\}$, and with this notation,$V^{*}\left(t_{k}\right)$ is the tumor at each phase. The following identity holds,

(1) $$\overset{9}{\underset{k=0}{⋃}}V^{*}\left(t_{k}\right)\subset V_{\mathrm{env}}$$

which is a direct consequence of $V^{*}\left(t_{k}\right)\in V_{\mathrm{env}}$, for all k.^20^ As this envelope is created by movement of a control volume, we can redefine it as,

(2) $$\begin{array}{c} V_{\mathrm{env}}=V_{\mathrm{ref}}^{*}\cup V_{\mathrm{SW}}^{*} \end{array}$$

where, $V_{\mathrm{SW}}^{*}$, is the *swept volume* produced by the movement of the reference which does not include the reference itself.^21^

#### 2.A.2 Removal of swept volume

To this end, generation of the tumor volume simply becomes a removal of the swept volume. To achieve this, we traverse $V_{\mathrm{env}}$ along the reverse motion path, $\gamma_{\chi }\left(t\right)\in \left[t_{90\%},t_{0\%}\right]$. We define $V_{\mathrm{env}}\left(t_{k}\right)$ as a shift in $V_{\mathrm{env}}$ so that $V_{\mathrm{ref}}^{*}$ takes up the position of $V^{*}\left(t_{k}\right)$. This ensures that $V_{\mathrm{ref}}^{*}$ is a subset of $V_{\mathrm{env}}\left(t_{k}\right)$ for all k, and as a result a subset of the intersection of all shifted envelopes,

(3) $$V_{\mathrm{ref}}^{*}\subset \overset{9}{\underset{k=0}{⋂}}V_{\mathrm{env}}\left(t_{k}\right)$$

obtaining a larger or equal volume regardless of shape, that is, $vol\left(\overset{9}{\underset{k=0}{⋂}}V_{\mathrm{env}}\left(t_{k}\right)\right)\geq vol\left(v_{\mathrm{ref}}^{*}\right)$.

Equality of the intersection and the reference volume can be demonstrated for convex shapes by contradiction. By Eq. (2), $vol\left(V_{\mathrm{env}}\left(t_{k}\right)\right)=vol\left(V_{\mathrm{ref}}^{*}\right)+vol\left(V_{\mathrm{SW}}^{*}\left(t_{k}\right)\right)$, where $V_{\mathrm{SW}}^{*}\left(t_{k}\right)$ is swept volume produced by movement of $V_{\mathrm{env}}$. As the envelope is larger in volume than the reference, the general expectation is that: $vol\left(V_{\mathrm{SW}}^{*}\left(t_{k}\right)\right)\geq vol\left(V_{\mathrm{SW}}^{*}\right)$ that is, a larger swept volume results from translating a larger control volume. With this view, the intersection would remove more volume than expected that is, $vol\left(\overset{9}{\underset{k=0}{⋂}}V_{\mathrm{env}}\left(t_{k}\right)\right)\geq vol\left(v_{\mathrm{ref}}^{*}\right)$, but by Eq. (3) volumes are equal. Due to overlap in positioning,

(4) $$\overset{9}{\underset{k=0}{⋂}}V_{\mathrm{env}}\left(t_{k}\right)=V_{\mathrm{ref}}^{*}$$

and the precise geometry can be obtained. This description shows it is possible to obtain tumor geometry from the intersection of the motion envelope shifted over the reverse motion path. All that remains is for this to be tested to determine if the approach is suitable for tumor shapes.

### 2.B Patients and 4D‐CT scans

To test the described theory, radiotherapy planning data were collected for 521 patients treated with SABR for a single lung lesion during 2011–2017 from an institutional archive. Patients were planned with 4D‐CT, and all respiratory phases and an iGTV contoured by a radiation oncologist in routine clinical practice (iGTV_obs_) were available.

Four‐dimensional CT scans were acquired using Philips Brilliance‐CT Big Bore Oncology® and Philips Bellows Device® to measure respiratory signal. Four‐dimensional data were sorted into ten respiratory bins of equal time 0%–90%, where 0% phase represents the inhale peak, and the exhale peak depends on individual breathing cycle.^22^ All scans were reconstructed to 512 × 512 image with slice thickness 3 mm, and most images have a square pixel size of 1.17 mm (range: 0.98 mm–1.37 mm). Approval was granted to collect and analyse patient data (REC reference: 17/NW/0060).

### 2.C Implementation of GTV generation

**Fig. 1:**
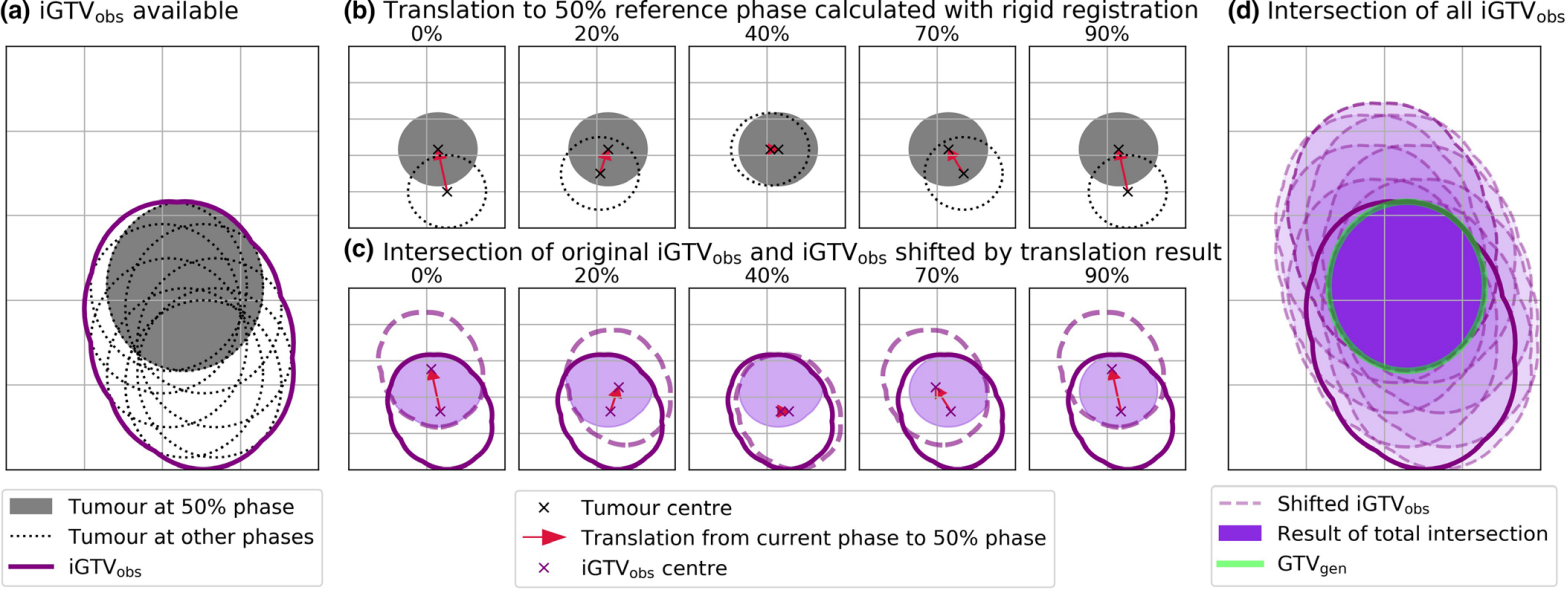
Two‐dimensional pictorial demonstration of the gross tumor volume (GTV) generation algorithm. (a) iGTV_obs_ approximates tumor volume union across all phases. (b) The translation required to map the tumor volume in each phase onto the reference phase (50%) is derived, with five phases of nine displayed. (c) The intersection of iGTV_obs_ and iGTV_obs_ translated by registration result forms the GTV edges. (d) The total intersection of all nine translated iGTV contours with the original approaches the GTV in the reference position (GTV_gen_).

Figure 1 summarises implementation of theory detailed in Section 2.A. On Fig. 1(a), iGTV_obs_ is the motion envelope ($V_{\mathrm{env}}$) formed by the reference volume ($V_{\mathrm{ref}}^{*}$) and the swept volume produced by its motion. In a clinical dataset, only iGTV_obs_ is available, we aim to remove the swept volume, so only the tumor volume (highlighted in gray) remains. The 50% phase was selected as reference, as this is near peak exhalation, which is considered the most stable position. As described previously, tumor motion is piecewise components that map the tumor position at 50% to every other phase. For all 521 patients, piecewise components were calculated from the 4D‐CT data using local rigid registration.

A volume of interest (VOI) to perform registration was defined as the iGTV_obs_ plus a 4mm spherical expansion. To produce optimal results, three adaptions on VOI were applied, leading to four possibilities:

1. VOI with no further adaptation,
2. VOI with removal of chest wall. To apply this, voxels with intensity greater than 176 HU were sampled to ensure bone and cartilage was detected, a closing operation was applied to connect structures, and the result subtracted from the VOI,
3. VOI with a prior assumed superior–inferior translation applied to each phase to follow an expected breathing motion of 2 cm peak‐peak, that is, to provide a better starting point for tumors moving more than 1 cm peak‐peak,
4. VOI with additional 6 mm expansion to 10 mm total, for mobile tumors that are difficult to distinguish from surrounding tissue.

For a given VOI, phase registrations were performed in a cyclic manner. After each registration, the next phase was prematched by applying the result of the previous phase. For each phase registration, the correlation ratio cost function was calculated, represented by a scale 0–1 where 1 is perfect matching.^23^ Mean and standard deviation (SD) of the cost function from all phase registrations was calculated. This was repeated for all VOI methods (1–4), and the method which produced the minimum SD for that patient was selected, under the assumption that the most successful registration provides the most consistent cost function.

The output was a translation set required to match the tumor inside the VOI on each phase to the reference, represented in Fig. 1(b). In practice, the translation encompasses movement in three directions superior–inferior (SI), anterior–posterior (AP), and left–right (LR).^24^ This is the reverse respiration path described in Section 2.A. Tumor motion amplitude was calculated by combining the difference in maximum and minimum position in all directions as a vector.

A mask of iGTV_obs_ was created and resampled to a 1mm slice thickness by nearest‐neighbor interpolation. Nine additional masks were formed by translating the iGTV_obs_ mask by the displacement required to map the tumor on each phase to the reference, setting up all shifted envelopes required for the intersection. Resampling allows for small SI shifts in translation to be considered. As demonstrated in Fig. 1(c), the intersection of each translated iGTV_obs_ with the original iGTV_obs_ defines borders of the GTV positioned in the reference phase. The intersection of all masks was sampled as a contour and is the generated GTV (GTV_gen_) [Fig. 1(d)]. As shown in Section 2.A, this approximates the tumor geometry, limited only by registration performance and quality of the iGTV_obs_.

### 2.D Comparison to manual delineation

Eleven patients that had considerable tumor motion were quasi‐randomly selected from the full cohort to provide variation in tumor location. The GTV was contoured by a radiation oncologist on the 50% phase (GTV_50%_) blinded to the previous iGTV_obs_. For comparison, surface distance‐to‐agreement (DTA) was calculated by extracting the absolute distance between each vertex on the surface of GTV_gen_ to the nearest vertex on the surface of GTV_50%_, with mean and standard deviation (SD) calculated across the surface. For geometric comparison, the volume of GTV_gen_ and GTV_50%_ was computed, and ratio calculated (GTV_gen_/GTV_50%_). To assess position, the vector distance between center of mass (CoM) of GTV_gen_ and GTV_50%_ was extracted.

### 2.E Application to full cohort

#### 2.E.1 Registration assessment

Gross tumor volume generation relies on registration accuracy. A visual registration rating of pass or fail was performed by a single observer who viewed a movie loop displaying a coronal slice at the iGTV_obs_ centre for each phase post registration. Fail was assigned if the tumor was not stable across all phases. Visual accuracy was compared against cost function mean and SD thresholds to assess whether failures could be automatically detected. Failed registrations were counted and removed from further analysis.

#### 2.E.2 Routine contour assessment

Gross tumor volume generation also relies on iGTV_obs_ quality. This method assumes that iGTV_obs_ reflects the tumor motion envelope, so, variation between iGTV_obs_ and expected envelope will affect GTV_gen_. The extent of this variation was detected by reversing the generation process to calculate the union of GTV_gen_ at every instance along the forward motion trajectory (iGTV_gen_). In the presence of variation, the border of iGTV_obs_ would not match the iGTV_gen_ border, and iGTV_gen_ is underestimated in comparison. To explain this, we note that GTV_gen_ was produced by the intersection of iGTV_obs_ shifted over the reverse trajectory, hence, the union of GTV_gen_ over the forward trajectory cannot be greater than the volume which produced it. Differences in shape propagated downstream will lead to underestimation in iGTV volume when propagated back as the border will not be accurately recreated (illustrated in Section S1 where the difference presents as concave perturbations across iGTV_gen_ surface). In general, the expectation is that larger underestimation in iGTV_gen_ compared to iGTV_obs_ will indicate a higher chance an error has occurred. To test for this, we extracted iGTV_gen_ and iGTV_obs_ volumes and calculated the iGTV volume ratio (iGTV_gen_/iGTV_obs_). If iGTV_obs_ and hence GTV_gen_ is accurate, iGTV volume ratio will be close to one. The DTA between surfaces was also extracted.

The lowest iGTV volume ratio that led to acceptable variation in the validation dataset was used to split the complete dataset into “check” and “trust” categories. An axial, coronal, and sagittal slice through the centre of each GTV_gen_ in the check group was viewed and subsequently rated as pass or fail. Any failures were removed from further analysis. The number of failures due to iGTV_obs_ quality was combined with the number of registration failures to give an overall measure of performance. Volume ratio was chosen as the assessment metric over surface DTA, as we do not expect a large difference in specific locations across the surface. Instead we aim to detect the combined impact of subtle edge differences and under‐estimation across the surface. Small differences across a surface adds to a detectable volume difference.

#### 2.E.3 Prognostic modelling

Clinical records of overall survival and patient demographics were collected for a subset of patients. The prognostic nature of iGTV_obs_ and GTV_gen_ volume was tested against overall survival with multivariable Cox regression. Clinical variables available were sex, age, T stage, performance status, comorbidity score, tumor lobe location, laterality, and histological sub‐type. Performance status (ECOG) is a grading 0 to 5 defined to describe a patient’s daily functioning ability, and comorbidity score (ACE‐27) a grading “None” to “Severe” based on pre‐existing medical conditions. A baseline clinical model was created using backward selection optimizing the Akaike Information Criterion (AIC). Three models were built for comparison: (a) clinical only, (b) clinical + iGTV_obs_, (c) clinical + GTV_gen_. An analysis of deviance was performed on nested models to identify which model produced a statistically significant change in performance: 1 vs 2, and 1 vs 3. In the analysis of deviance, the chi‐square is calculated by subtracting the deviance (−2 log‐likelihood) of the updated model from the clinical model. A higher chi‐square is representative of a larger reduction in deviance, hence improved model fit. If both models produced a significant change, the optimum model was the model with the lowest AIC. All statistical analysis was performed in R version 3.5.2.

## 3 RESULTS

### 3.A Comparison to manual delineation

For the validation subset, the mean tumor motion amplitude was 7.47 mm, range 2.75 to 12.6 mm. Registration performed well on visual assessment and had mean cost 0.95. Patients analyzed were of T stage 1 or 2, with five upper lobe and six lower lobe located tumors. Patients analyzed are labeled 1–11, with characteristics recorded in Table S1. The CPU time taken to generate a GTV per patient from loading data to output was 35 s (including approximately 25 s for registration) using nonoptimised software and dual Intel® Xeon® processors (2.20 Ghz). Table  I displays comparison results for GTV_gen_ vs GTV_50%_ contour.

**Table I:** Table of validation results comparing GTV_gen_ to GTV_50%_. DTA: Surface perpendicular distance‐to‐agreement. SD: Standard deviation. CoM: Centre of mass.

Patient	GTV50% volume (cc)	GTVgen volume (cc)	Mean DTA (mm)	SD DTA (mm)	CoM distance (mm)
1	4.51	5.83	1.08	0.90	0.50
2	3.16	1.58	1.09	0.86	1.94
3	3.61	2.92	0.79	0.74	0.90
4	2.76	3.37	1.06	1.02	0.91
5	9.05	6.98	0.89	0.84	3.38
6	16.06	11.49	1.35	1.03	0.85
7	2.57	3.93	1.55	1.51	1.92
8	6.83	5.37	0.79	0.78	0.87
9	14.42	12.24	1.01	0.92	0.94
10	7.86	4.89	1.16	0.91	0.92
11	4.32	3.46	0.80	0.68	0.21
*Mean*	*6.83*	*5.64*	*1.05*	*0.93*	*1.21*
*SD*	*4.68*	*3.42*	*0.24*	*0.22*	*0.89*

The generated contour agreed with an expert contour with an average mean DTA of 1.05 mm and SD DTA of <1 mm, comparable to pixel spacing. The GTV volume ratio across all patients was 0.5–1.53, which is approximately 50% over or under‐estimation for the most extreme cases. For both these cases (2 and 7), this results in a <1.6 cm^3^ volume difference. The largest volume difference reported is 4.57 cm^3^. In majority of cases volume is underestimated (73%). Ten patients had positional error of <2 mm difference in CoM, with eight meeting a <1 mm criteria.

**Fig. 2:**
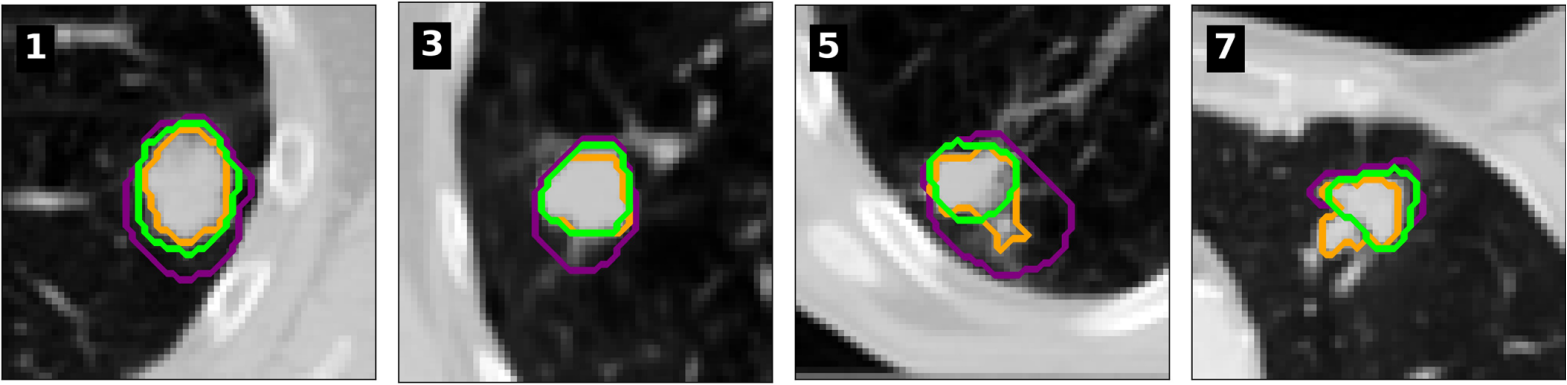
The iGTV contour (purple), radiation oncologist’s gross tumor volume (GTV) contour on the 50% phase, GTV_50%_, (orange), and generated GTV, GTV_gen_, on the 50% phase (lime) for patients 1) representative of average performance, 3) representative of good performance, 5) largest positional shift, and 7) largest distance‐to‐agreement.

Figure 2 provides a single slice visual example of four patients. Patient 1 was selected to be representative of the average performance, and patient 2 for the best‐case scenario, in both the GTV_gen_ contour closely matches expert opinion. Patient 5 performed worst on positional accuracy; this was caused by sub‐solid tumor extensions not visible on all phases included in GTV_50%_ but not GTV_gen_. Patient 7 had worst DTA results due to a nodule included in GTV_50%_ and not iGTV_obs_, hence not included in GTV_gen_.

### 3.B Full cohort results

#### 3.B.1 Registration assessment

Across all 521 patients, a mean tumor motion amplitude 6.78 mm was measured, range 0 to 36.2 mm. The mean iGTV_obs_ volume was 9.71 cm^3^, range 0.35 to 73.3 cm^3^. Registration performed well with average and SD of mean cost function across phases 0.92 and 0.06, respectively, for the selected registration method.

On visual assessment, 23 patients (4.4%) were recorded as registration failures. Seven of 23 patients had complete generation failure with no contour produced. All complete generation failures had iGTV_obs_ volume below mean patient volume (less than 8cm^3^), and all had >22 mm tumor motion amplitude estimated from incorrect registration. Reasons for failure are detailed in Table S2.

**Fig. 3:**
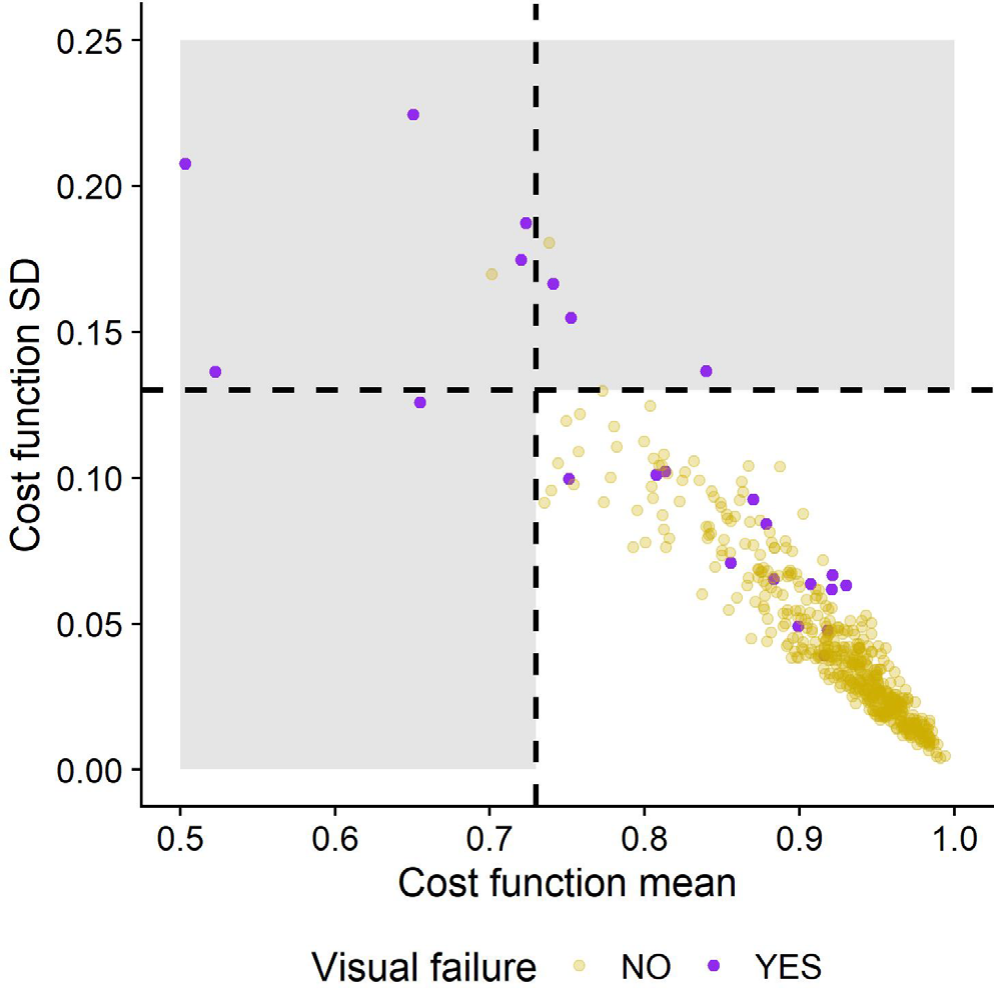
Plot displaying results of registration assessment. The mean and standard deviation of the cost function for optimal method is displayed, with data points colour coded to show result of visual assessment. Dashed lines display thresholds on registration based on these data.

Registration accuracy was not fully described by SD and mean cost as this did not include all visual assessments, as depicted in Fig. 3. Overall, we removed patients that failed on visual assessment, or had a mean cost <0.73, or SD of the cost function greater than or equal to 0.13. 25 patients (4.8%) were removed with this assessment (i.e., all points within the shaded background or highlighted in purple), leaving 496 patients for remaining analysis.

#### 3.B.2 Routine contour assessment

In the validation group, the iGTV volume ratio range was 0.84 to 0.97. DTA SD and mean range was 0.05–0.53 mm and 0–0.19 mm, respectively. Across 496 remaining patients, the average volume ratio was 0.93 with range 0.52 to 1.06. Some tumors have a ratio slightly greater than one, driven by a few border pixels. This is due to the interpolation used in generation allowing partial voxels to be included when compared to a contour from the original image spacing. However, these are not of concern due to the small volume difference (1.06 maximum ratio) which does not impact the GTV quality.

**Fig. 4:**
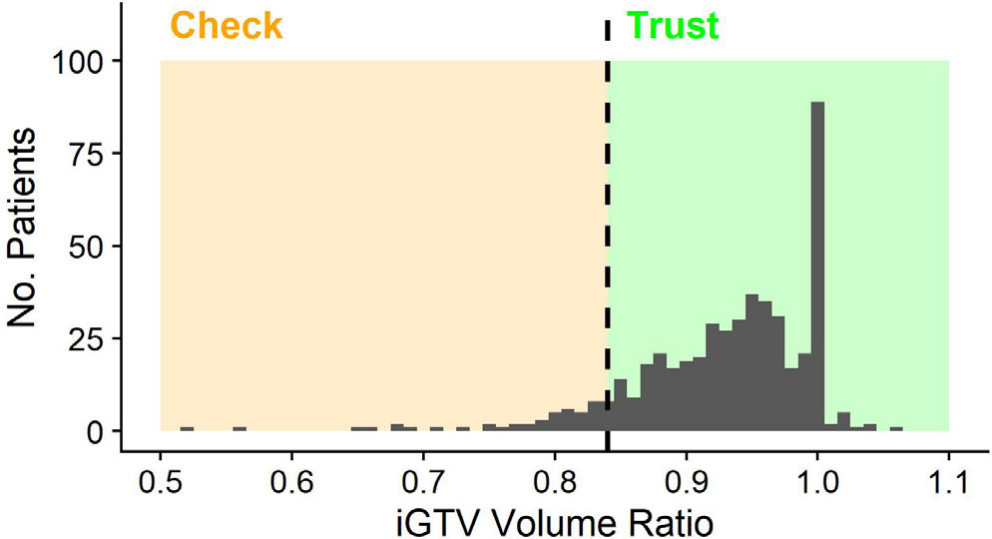
Histogram of iGTV volume ratio for all patients. Orange “check” region demonstrates cases that could be triggered for visual assessment, green “trust” region demonstrates those that could pass without assessment.

A histogram of iGTV volume ratio for these patients is displayed in Fig. 4, with markers demonstrating the “check” and “trust” category. The limit set was taken as the smallest value in the manual validation dataset: 0.84. In the full dataset, 42 patients fall below this threshold. On visual assessment of this group, only five were rated as failures and removed from analysis, example contours are displayed in Section S3.B. Overall, there was a 94% success rate from all assessments performed.

#### 3.B.3 Prognostic model performance

After registration and contour assessment, 491 patients remained of which 402 had clinical records on overall survival and patient demographics available (reported in Table S4). Histology was not considered as there was 56% missing data, but all other clinical variables were included. Three hundred and eight patients had complete information for all remaining variables, in which there were 119 events. Level 1 was selected as reference for performance status, and “Mild” for the comorbidity score, due to low patient number in the first levels. This does not impact analysis but warrants caution when interpreting regression coefficients.

Following feature selection, only the comorbidity score was selected for the clinical model (Table S5). Table II details multivariable analysis results. Both iGTV_obs_ and GTV_gen_ improved the clinical model with a significant reduction in deviance (χ^2^(1)), (12.7, *P* < 0.001) and (15.4, *P* < 0.001) respectively. Overall, GTV_gen_ produced the lowest AIC.

**Table II:** Multivariable analysis of a clinical model, and two further models with a volume variable included. HR: hazard ratio; 95% CI: 95% confidence interval.

	1) Clinical		2) Clinical + iGTVobs		3) Clinical + GTVgen	
	HR (95% CI)	*P* value	HR (95% CI)	*P* value	HR (95% CI)	*P* value
ln(iGTV_obs_ volume)			1.43 (1.17–1.74)	<0.001		
ln(GTV_gen_ volume)					1.43 (1.19–1.72)	<0.001
Comorbidity score (Mild reference)						
None	0.94 (0.28–3.18)	0.918	1.06 (0.31–3.60)	0.925	1.08 (0.32–3.66)	0.906
Severe	1.64 (0.96–2.81)	0.073	1.58 (0.92–2.70)	0.098	1.55 (0.90–2.66)	0.113
Moderate	2.38 (1.41–4.04)	0.001	2.22 (1.31–3.77)	0.003	2.22 (1.31–3.77)	0.003
*AIC*	*1194.99*		*1184.27*		*1181.61*	

## 4 DISCUSSION

In this article, we formally describe, implement, and validate a new method for generating the GTV from routine iGTV contours on 4D‐CT data. This method had a 94% success rate on a clinical dataset. Success was defined as excellent registration accuracy, and iGTV volume ratio greater than or equal to 0.84 or iGTV volume ratio below 0.84 but contour closely matched with tumor on visual assessment. This method is not intended for clinical purpose, but for retrospective generation of the GTV and it will aid analysis of tumor radiomic features and dose parameters on routine data without the need for time‐consuming manual delineation that has been favoured in current research.^15^ This technique can be implemented with any automated registration algorithm allowing it to be efficiently applied to data at any institution that adopts an iGTV planning approach, with a fully automated result in approximately 35 s per patient.

As we have formally described the methodology, the potential limitations to consider are:

1. how well the observer delineated iGTV describes the tumor volume union, and,
2. how well registration describes tumor motion.

To show feasibility for large datasets, we demonstrated techniques for detecting these failures that reduces the need for visual assessment. Registration checks could be targeted by location, as although infrequent, failed registrations were due to small tumors near the diaphragm. Contour assessment could be targeted by iGTV volume ratio as it detects cases with more variation than expected (Fig. S5).^25^ Like location for registration, this ratio is intended to guide visual assessment depending on the criteria required and is not a fixed rule. The threshold implemented for checks can be adapted to balance contour accuracy and time available for visual assessment. With further study, this ratio may be useful for clinical iGTV quality assessment, as a simple tool that can be extracted in many softwares. As MIP scans can underestimate tumor volume, a quality check is beneficial, especially for the 35% of UK centres using such a technique without systematically checking the coverage on individual phases.^10^

For 308 NSCLC patients treated with SABR, additional prognostic information on overall survival was provided by GTV_gen_ volume compared to the iGTV_obs_ volume. Although no model performed highly and potentially prognostic clinical variables were not included in the multivariable model (i.e. histological sub‐type,^26^ or performance status^27^), the purpose was comparison rather than development of a clinical prediction model. This provides confidence in using the generated volume as the tumor volume covariate in statistical modelling. It is important to accurately model volume, as although inconclusive,^28^ tumor volume is often prognostic in the SABR setting.^29^, ^30^, ^31^ It is also important to control for tumor volume in radiomics analysis as it is often a confounding factor.^32^, ^33^

For validation, we used manual delineation by an expert which is the current gold standard for auto‐contouring studies. In the validation set, GTV_gen_ agreed with an expert contour with an average mean DTA 1.05 mm, and SD DTA 0.93 mm. This is within the range of observer variability for early‐stage NSCLC, reported as 1.2–1.8 mm by Peulen et al.,^34^ and 1.5 mm in the transverse plane and 2.6 mm in the SI direction by Persson et al.^35^ It is important to note a distinct difference in our DTA analysis. To quantify local delineation variability we reported an absolute DTA between two contours, however, the standard deviation of DTA from all contours to the median is reported by Peulen and Persson^34^, ^35^ as described by Steenbakkers et al.^36^ Overall, results demonstrate that shape has been accurately produced. In addition, difference in GTV volume is well within observer variation. Average differences of 6 cm^3^ have been reported, with a maximum of 18 cm^3^.^35^ The maximum difference observed in this study was <5 cm^3^.

The majority of validation cases met ICRU criteria of <2 mm positional accuracy.^37^ Interestingly, decreased geometric and positional accuracy for individual cases occurred due to issues with iGTV_obs_ contour as opposed to algorithm performance. Patient 5 displayed worst positional accuracy, with a 3.38 mm CoM difference. On visual inspection, a low‐density tumor extension was visible on the 50% phase and not the inhalation phase. Therefore, the GTV_50%_ shape was not comparable to the visible tumor on all phases, so, iGTV_obs_ did not represent the GTV_50%_ union. Patient 7 displayed the worst DTA with 1.55 mm mean, and 1.51 mm SD. This was due to observer disagreement about a nodule included in GTV_50%_ but not iGTV_obs_. The observer participating in this study commented they would not have agreed with the iGTV_obs_ when looking in retrospect.

Generalisation of the validation results to the larger dataset is valid due to comparable registration performance with mean cost 0.95 for validation, and 0.92 for the full cohort. Although different registration methods were implemented, there was no difference in performance for patients with different methods implemented. This was a purely a tool to best account for the motion trace. All methods are sensitive to single phase registration failures, which could occur as a result of an artefact or reduced tumor visibility. For tumors with little hysteresis, problem phases could be excluded, as only a few phases are required to produce an accurate GTV shape representation from the iGTV. Another influence on registration success is the choice of reference phase. In this study we chose 50%, but any phase could be chosen, or the motion curves from different reference phases could be averaged.^38^

It is clear our automated tool will not alleviate the issue of observer variability or registration error, and the visual assessment guidance is not intended as a fully validated tool for detecting all failures. However, the technique allows for a quick, reproducible alternative to retrospective manual contouring. Of course, an alternative method would be to perform auto‐segmentation on the 50% phase directly. However, published automated GTV generation methods show limited success with small lung tumors, due to hazy appearance and uncertain boundaries, with 50% of cases requiring substantial manual adjustment.^39^ Alternatively, machine‐learning techniques have been investigated, however, these require large cohorts of accurate labelled training data that are representative of a range of tumor types.^40^ Typically derived from a single institution, machine learning techniques require multi‐institutional data to guarantee applicability. With our technique, training data is not required, and a registration algorithm can be easily adapted to work well on a range of tumor types, as shown with our implementation of four registration methods.

Another advantage to this method is that it can directly incorporate information from the iGTV used for treatment planning. Differences between clinician drawn contours and auto‐segmentation approaches are thought to be important for modelling treatment outcome.^41^ In studies where such variability is important to consider or in radiomics, where contour accuracy is of high importance, potential suggestions would be to test a combination of automated and manual approaches, or produce a consensus from several methods.^42^ Alternatively, the method presented in this paper for detecting delineation uncertainties could be used to apply a population‐based expansion of the contour to shift the iGTV ratio close to one to help ensure tumor coverage. Similarly, contour perturbation techniques could be considered to account for uncertainty in the modelling process.^43^

Our technique is the second of its kind proposed in the literature to extract a GTV from the iGTV. We believe the two techniques complement each other, and consideration should be made on which approach to adopt depending on the dataset and purpose. The method developed by Johnson et al was validated in 15 patients to provide accurate volume estimates for larger volume tumors, but was not consistently validated for shape sensitive parameters (i.e., DTA) so the development of a method for shape estimation was encouraged.^16^ We have developed a technique that provides accurate volume and boundary estimates for early‐stage lung tumors, not typically attached to rigid structures or invading mediastinum.^44^ The applicability of this method for large tumors requires further validation.

## 5 CONCLUSIONS

In summary, we have developed a method to automatically produce a GTV using a combination of pre‐existing expert iGTV contour and local rigid registration of the tumor on 4D‐CT data. This allows for the GTV to be estimated with no training data required, accurate within the range of expected observer variation. This technique will facilitate the study of GTV characteristics on all phases of 4D‐CT data. Finally, we displayed applicability of this technique to a large clinical dataset by developing approaches to reduce time‐consuming visual checks.

## CONFLICT OF INTEREST

The authors have no conflict of interest to declare.

## Supporting information


**Data S1**. Supplementary information.
