# Supplementary material for: Automated gross tumor volume contour generation for large‐scale analysis of early‐stage lung cancer patients planned with 4D‐CT
Source: Med Phys. 2020 Dec 30;48(2):724–32. doi: 10.1002/mp.14644 (PMC7986204; doi:10.1002/mp.14644)
Supplement: Supplementary file 1 — Data S1. Supplementary information. [file MP-48-724-s001.pdf]

Automated gross tumour volume contour generation for large-scale analysis of early-stage lung cancer patients planned with 4D-CT

Angela Davey, Marcel van Herk, Corinne Faivre-Finn, Sean Brown, and Alan McWilliam

Supplementary Material

1 Routine contour assessment

The main assumption in our method is that the $iGTV_{obs}$ delineated on the MIP scan represents the GTV union across all phases. Figure 1 depicts an example where $iGTV_{obs}$ does not match this ideal situation.

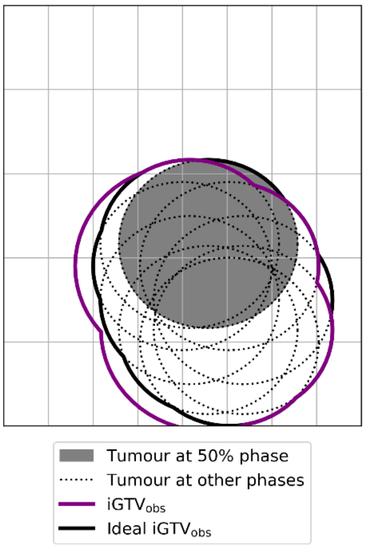

Figure 1. In this pictorial example $iGTV_{obs}$ (purple) does not match the union of GTV volume across all phases (black), there is overestimation on the left, and underestimation on the right.

This variation causes differences in GTV_{gen} when our method is applied, showcased in Figure 2.

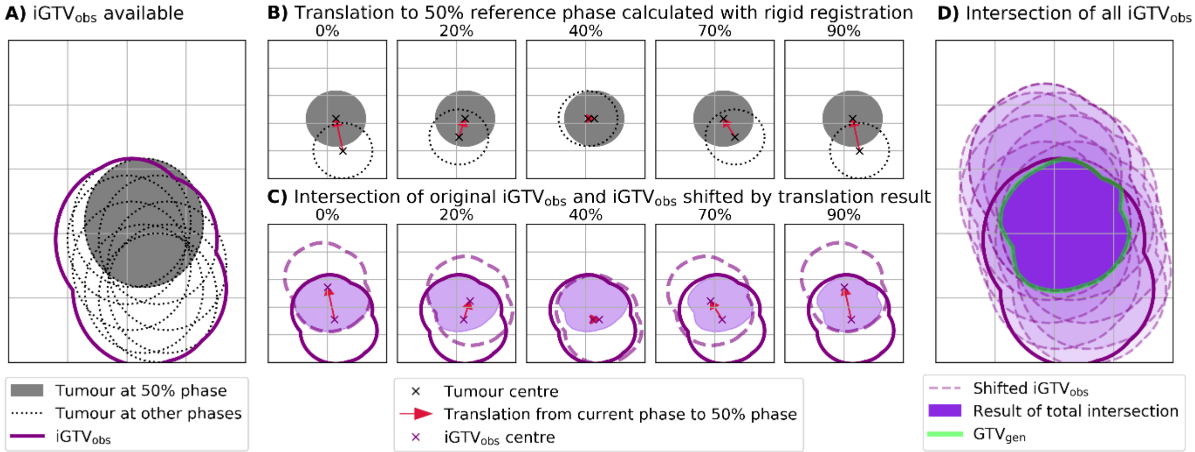

Figure 2: Repeat of GTV generation demonstration. A) $iGTV_{obs}$ *roughly* approximates tumour volume union across phases. B) Translation required to map tumour on each 4D-CT phase to the reference. C) Intersection forms GTV edges. D) Intersection of all translated $iGTV_{obs}$ contours. The impact of variability in $iGTV_{obs}$ has presented itself with difference in GTV_{gen} shape compared to tumour shape.

Variation is detected by reversing the generation process to form an $iGTV_{gen}$. To implement this, GTV_{gen} is shifted over the forward motion trajectory and the union of all shifted GTV_{gen} is calculated. As the variation in shape caused by the $iGTV_{obs}$ is present in GTVgen, the union would not regenerate the

exact shape of iGTV$_{obs}$, as shown in Figure 3. Here we see that perturbation in the contour presents as an underestimation in volume. Note, an underestimation in volume will also be detected if there is underestimation in motion, but this likelihood is reduced on registration assessment.

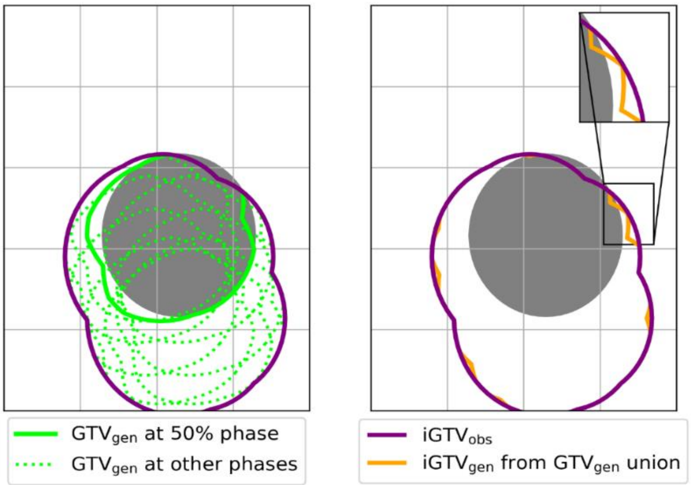

Figure 3: Pictorial demonstration of the detection of observer variation in iGTV$_{obs}$ (purple) compared to iGTV$_{gen}$ created from the GTV$_{gen}$ union (orange).

2 Validation analysis

Patient characteristics and registration result for the validation set are detailed in Table 1.

Patient	T-stage	Location	Motion amplitude (mm)	Mean Cost	SD Cost
1	2a	LLL	7.20	0.94	0.03
2	1b	RUL	7.95	0.94	0.03
3	1a	RUL	9.24	0.96	0.02
4	1a	LLL	7.56	0.95	0.03
5	2a	RLL	9.60	0.94	0.04
6	2a	RUL	7.71	0.93	0.03
7	1a	LUL	2.75	0.95	0.03
8	1b	RLL	12.60	0.93	0.04
9	2a	LLL	5.06	0.94	0.04
10	2a	RUL	4.43	0.95	0.03
11	1a	RLL	8.08	0.98	0.01
Mean			*7.47*	*0.95*	*0.03*
SD			*2.68*	*0.01*	*0.01*

Table 1. Patient information for validation data, along with the tumour motion amplitude calculated from registration, and the mean and standard deviation (SD) cost for registration across phases. LLL: left lower lobe, RLL: right lower lobe, LUL: left upper lobe, and RUL: right upper lobe.

3 Full cohort analysis

3.1 Registration assessment

Registration was implemented using all four methods across patient set: method one for 146 patients, two for 76, three for 256, and four for 43. There were seven cases (1.3%) which failed to generate a contour. All these cases failed on visual assessment of registration, despite some displaying high cost mean and low standard deviation. The reasons for these failures are detailed in Table 2.

$iGTV_{obs}$ volume (cc)	Tumour motion amplitude (mm)	Mean cost	SD cost	Failure reason
1.28	27.0	0.92	0.04	Close to diaphragm
2.50	35.0	0.90	0.05	Close to diaphragm
4.67	22.0	0.75	0.10	Close to mediastinum
4.78	27.1	0.88	0.08	Close to diaphragm
7.95	32.4	0.84	0.14	Close to diaphragm
6.76	36.2	0.52	0.14	Atelectasis near tumour region
4.42	29.3	0.65	0.13	Close to chest wall

Table 2. Additional detail on patients for which the method did not generate any contour.

Two examples of cases which led to registration failure are shown in Figure 4.

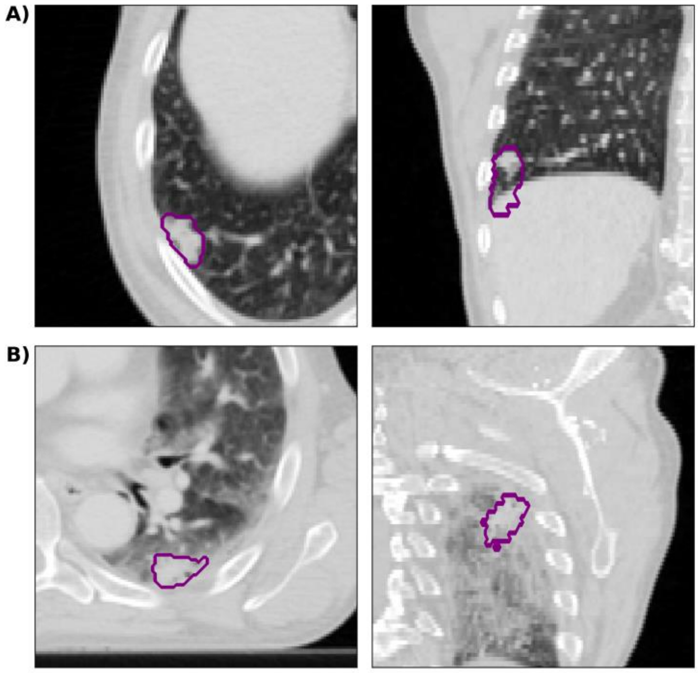

Figure 4 Examples of patients with registration failures with axial (left) and coronal (right) view and $iGTV_{obs}$ shown in purple. Issues caused by **A)** diaphragm invasion in VOI and, **B)** atelectasis around the tumour.

3.2 Contour assessment

The iGTV volume ratio for validation cases are shown in Table 3.

Patient	$iGTV_{obs}$ Volume (cc)	$iGTV_{gen}$ Volume (cc)	iGTV Volume Ratio
1	10.37	9.63	0.93
2	3.72	3.12	0.84
3	6.41	5.81	0.91
4	5.52	5.20	0.94
5	15.22	12.77	0.84
6	18.47	17.69	0.96
7	5.39	5.17	0.96
8	12.62	11.37	0.90
9	16.55	15.83	0.96
10	7.97	7.76	0.97
11	6.38	6.02	0.94
Mean	*9.88*	*9.12*	*0.92*
SD	*5.09*	*4.76*	*0.05*

Table 3. Table showcasing $iGTV_{obs}$ variation in the validation dataset.

Based on the threshold of the lowest value in this validation set, 42 contours were assessed visually. These patients display an unexpected amount of variation as shown in Figure 5.

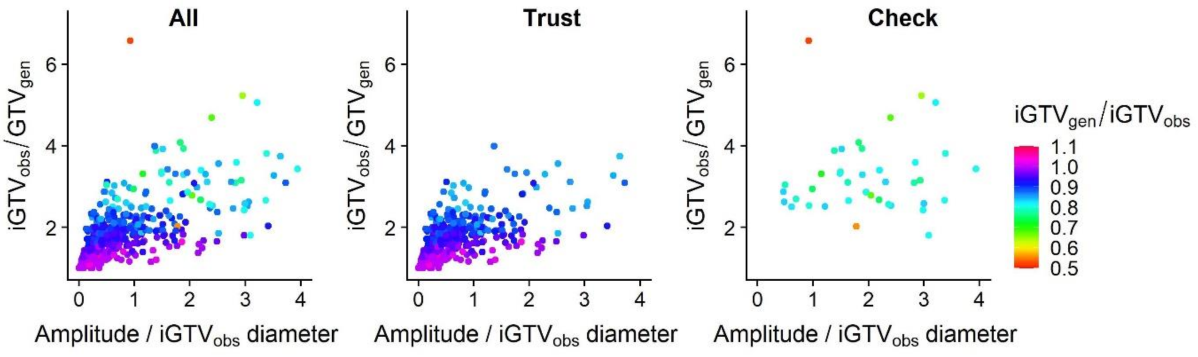

Figure 5. Comparing iGTV volume ratio against the natural expected relationship between iGTV, GTV and motion (Mercieca et al, 2018), for all patients, the trust group, and the check group (left to right).

As an example, for 5 failures and 5 pass examples the centre axial slice and GTV_{gen} are shown in Figure 6, and the centre coronal slice Figure 7. This rating is subjective but is an example of how this technique can be implemented.

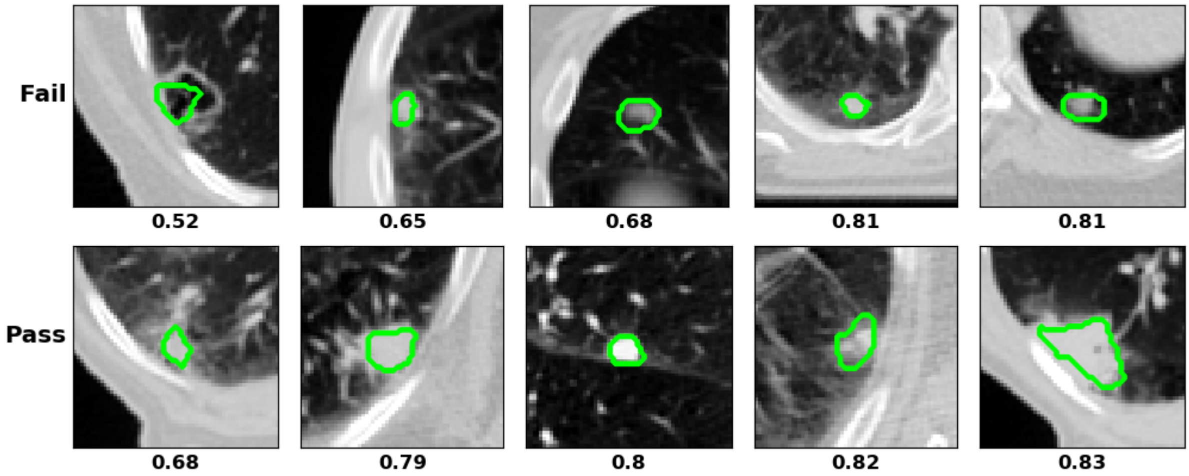

Figure 6. *Top:* Axial slice of the five cases rated as failures with the ratio recorded. *Bottom:* five examples of passes with ratio recorded.

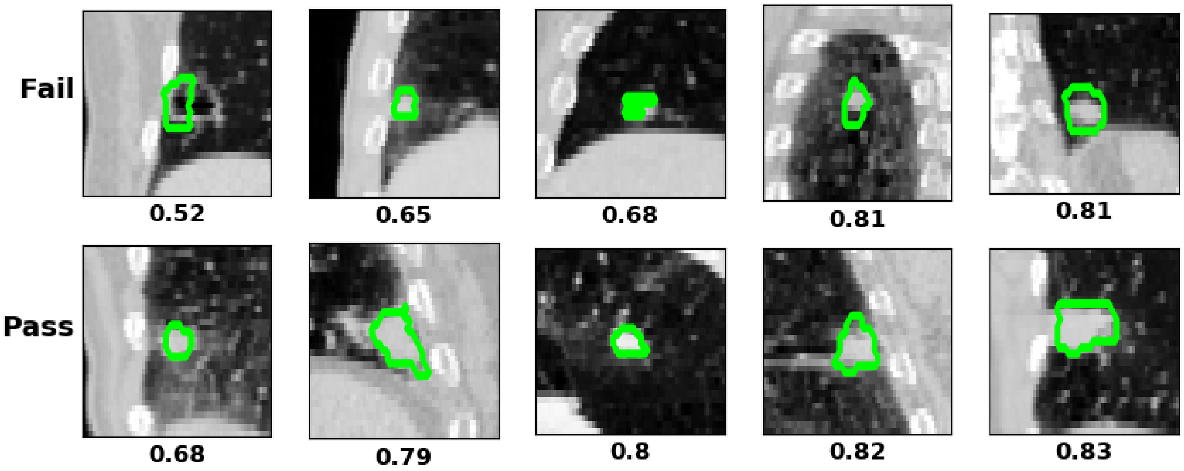

Figure 7. *Top:* Coronal slice of the five cases rated as failures with the ratio recorded. *Bottom:* five examples of passes with ratio recorded.

4 Patient data

Patients with clinical data available for survival analysis are detailed in Table 4. For lobe location, any recorded as ‘middle’ has been combined with upper for ease of comparison.

**Variable**	**Categories**	**Number**	**Total Number**
Sex	Female	200	402 (100%)
	Male	202	
Age	Median (Range)	75 (45 - 93)	402 (100%)
T Stage	T1	234	366 (91%)
	T2	131	
	T3	1	
Performance status (ECOG)	0	7	355 (88.3%)
	1	111	
	2	185	
	3	52	
Comorbidity score (ACE-27)	None	10	317 (78.9%)
	Mild	69	
	Moderate	112	
	Severe	126	
Tumour lobe location	Lower	126	394 (98%)
	Upper	268	
Tumour laterality	Left	146	394 (98%)
	Right	213	
Histological subtype	Adenocarcinoma, NOS	72	177 (44%)
	Squamous cell carcinoma, NOS	68	
	Carcinoma, NOS	28	
	Other	9	
$iGTV_{obs}$ volume (cm^3)	Median (Range)	7.37 (0.59 - 73.3)	402 (100%)

Table 4. Clinical information available to build a survival model. Total number is based on the proportion of the 402 patients with outcome data that have values available for those categories.

The AIC at each step of the backward selection process is detailed in Table 5.

**Step**	**AIC**
Full model	1205.54
- Tumour lobe location	1203.55
- Tumour laterality	1201.57
- Performance status	1199.60
- Motion amplitude	1197.68
- T stage	1195.97
- Sex	1195.12
- Age	1194.99

Table 5. Results of backward selection to produce the optimum clinical model for comparison purpose.
